# Acute T-Cell Rejection after Living-Donor Kidney Transplantation: Monitoring with Urinary Presepsin

**DOI:** 10.7759/cureus.95462

**Published:** 2025-10-26

**Authors:** Kumiko Fujieda, Akihito Tanaka, Takaya Ozeki, Kazuhiro Furuhashi, Yuta Sano, Shohei Ishida, Shoichi Maruyama

**Affiliations:** 1 Department of Nephrology, Nagoya University Hospital, Nagoya, JPN; 2 Department of Urology, Nagoya University Hospital, Nagoya, JPN; 3 Department of Nephrology, Nagoya University Graduate School of Medicine, Nagoya, JPN

**Keywords:** kidney transplant, presepsin, renal function, t-cell-mediated rejection, urinary biomarkers

## Abstract

Rejection remains a major complication after kidney transplantation, yet biopsy carries risks and delays. Therefore, non-invasive biomarkers are needed to monitor rejection.

The patient was a 27-year-old man who was diagnosed with focal segmental glomerulosclerosis one year earlier. His renal function gradually deteriorated, and he was referred to our hospital for living-donor kidney transplantation, which was performed with his mother as the ABO-compatible donor. A standard immunosuppressive protocol was administered with steroids, basiliximab, mycophenolate mofetil (MMF), and tacrolimus. On postoperative day 5, he contracted COVID-19 and was treated with molnupiravir; however, antigen positivity persisted, leading to a reduction in MMF. The serum creatinine level reached a minimum of 2.41 mg/dL on postoperative day 7 but subsequently rose to 5.86 mg/dL. An episode biopsy revealed acute T cell-mediated rejection (TCMR). He was treated with steroid pulse therapy and anti-thymocyte globulin, after which his renal function stabilized. Protocol biopsies at three months and one year also demonstrated improvements in TCMR. Urinary presepsin levels at the time of TCMR and at the three-month and one-year protocol biopsies were 13,075, 1,332, and 680 ng/g creatinine, respectively, correlating with changes in renal function and histological findings.

This case suggests that urinary presepsin may be a valuable noninvasive biomarker for monitoring disease activity in TCMR.

## Introduction

Early diagnosis of rejection in kidney transplant recipients is critical for the timely initiation of therapy and preservation of graft function. Although renal biopsy remains the gold standard for diagnosing rejection, it is invasive and carries risks such as bleeding, arteriovenous fistula, and infection [1,2]. Furthermore, definitive results may take several days, limiting their utility for real-time monitoring. These limitations highlight the need for novel, non-invasive biomarkers that could serve as alternatives to renal biopsy and allow for continuous monitoring.

To date, various noninvasive biomarkers have been investigated for the diagnosis and prediction of kidney transplant rejection. For example, measurements of urinary CXCL9 and CXCL10, as well as diagnostic assays combining multiple urinary DNA, protein, and metabolic biomarkers, have been reported [3-6]. However, the clinical utility and predictive performance of these biomarkers in real-world settings have not been fully validated, and challenges such as standardization of sample measurement remain, limiting their application in routine clinical practice.

Presepsin, a soluble CD14 subtype, has emerged as a promising biomarker. It is predominantly secreted by monocytes following phagocytosis. Serum presepsin levels increase during the early stages of sepsis and correlate with disease severity [7,8]. The presepsin assay is currently implemented in clinical practice and utilized for the early diagnosis and monitoring of sepsis. Our research group previously demonstrated that urinary presepsin reflects inflammatory cell infiltration in the renal interstitium [9]. In addition, we reported that urinary presepsin serves as a useful marker for detecting T-cell-mediated rejection (TCMR) in kidney transplant recipients [10].

In this report, we describe a case in which the reduction of immunosuppressive therapy following COVID-19 infection precipitated TCMR. The case highlights the potential utility of urinary presepsin not only for diagnosing TCMR but also for longitudinal assessment of disease activity.

## Case presentation

The patient was a 27-year-old male who, in 2022, had impaired renal function (serum creatinine 2.67 mg/dL) and proteinuria. A renal biopsy performed at another hospital revealed focal segmental glomerulosclerosis (FSGS). At that time, he was obese (BMI 30.8 kg/m^2^) and had poorly controlled diabetes mellitus (HbA1c 17.2%), and secondary FSGS due to metabolic abnormalities was suspected. Steroid therapy was considered likely to worsen his diabetes; therefore, conservative management was chosen. Renal function gradually declined, and in April 2023, he was referred to our hospital for living-donor kidney transplantation. He had multiple comorbidities, including hypertension, hyperuricemia, type 2 diabetes mellitus, dyslipidemia, and obesity, and was taking multiple medications to manage them. His height and weight were 156 cm and 75 kg (BMI 30.8 kg/m²), respectively, and his blood type was A. Vital signs at the initial visit were temperature 36.4℃, blood pressure 140/95 mmHg, pulse 83 beats/minute, respiratory rate 12 breaths/minute, and SpO₂ 99% on room air. Mild lower leg edema was noted; however, no other abnormalities were observed on physical examination.

The donor, his mother, was 153 cm tall, weighed 54 kg (BMI 23.1 kg/m²), and had blood type A. Her medical history included asymptomatic cerebral infarction, hypertension, and hyperlipidemia. Human leukocyte antigen (HLA) typing revealed three mismatches. All direct crossmatch results were negative for warm T, warm B, and cold B cells, and flow cytometry crossmatch was also negative for both T and B cells. Anti-HLA antibody screening was 0% for classes I and II.

Seven months after the initial evaluation, the patient was admitted for kidney transplantation. Laboratory results at admission are summarized in Table 1, and urinalysis results are presented in Table 2. The C-reactive protein (CRP) level was elevated at 6.05 mg/dL, attributed to a gout attack. During the pre-transplant period, the patient developed fluid overload; a dialysis catheter was inserted, and he underwent four sessions of hemodialysis.

**Table 1:** Results of the patient's blood test on the first visit

Parameter	Patient values
White blood cell count (/μl)	9200
Red blood cell count (10^6^/μl)	3.35
Hemoglobin (g/dl)	9.0
Platelet count (10^4^/μl)	31.0
Glucose (mg/dL)	82
Total protein (g/dL)	6.7
Albumin (g/dL)	3.3
Blood urea nitrogen (mg/dL)	66.7
Creatinine (mg/dL)	8.84
Uric acid (mg/dL)	8.4
Sodium (mmol/L)	138
Potassium (mmol/L)	4.5
Chlorine (mmol/L)	107
Calcium (mg/dL)	6.7
Phosphorus (mg/dL)	5.7
C-reactive protein (mg/dL)	6.05

**Table 2:** Results of the patient's urine test on the first visit HPF: high power field

Urinalysis	Patient values
Proteinuria	3+
24-hour urine protein excretion	5.7 g/day
Occult blood	+
Red blood cells	1-4/HPF
Leukocytes	-
White blood cells	1-4/HPF
Granular casts	+
Hyaline casts	+
Fatty casts	+

**Figure 1:**
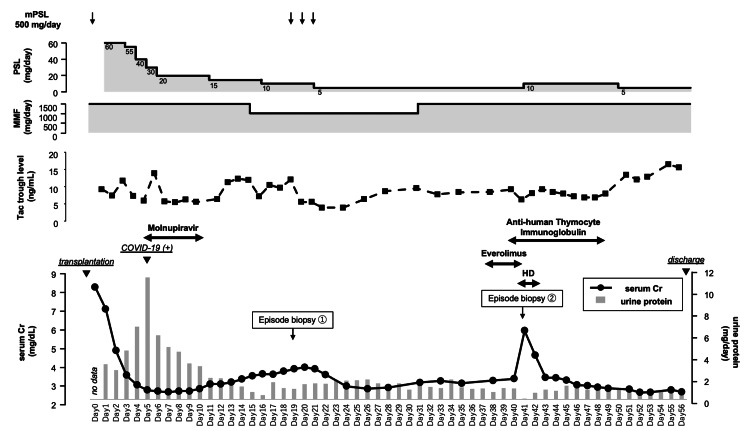
The patient's clinical course during hospitalization Serum creatinine and urinary protein levels are shown throughout hospitalization, alongside the timing of immunosuppressive and supportive therapies. PSL: prednisolone; mPSL: methylprednisolone; MMF: mycophenolate mofetil; Tac: tacrolimus; Cr: creatinine; HD: hemodialysis

**Figure 2:**
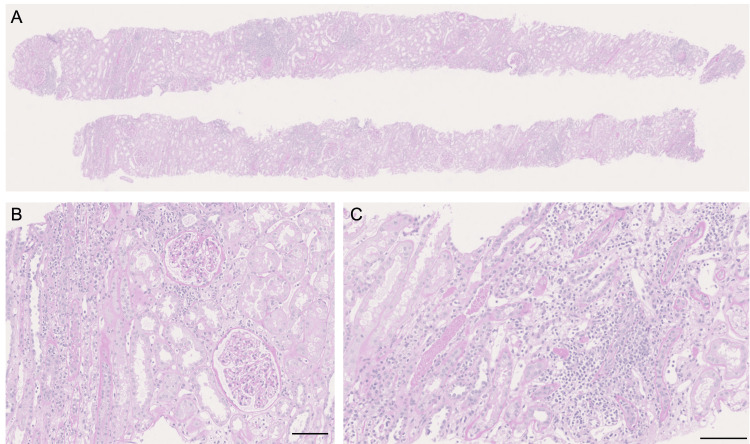
Histological findings of the first episode biopsy (A) Low-power view of the entire section; (B) Tubulitis; (C) Interstitial inflammatory cell infiltration, periodic acid-Schiff (PAS)-staining; scale bar =100 μm

**Figure 3:**
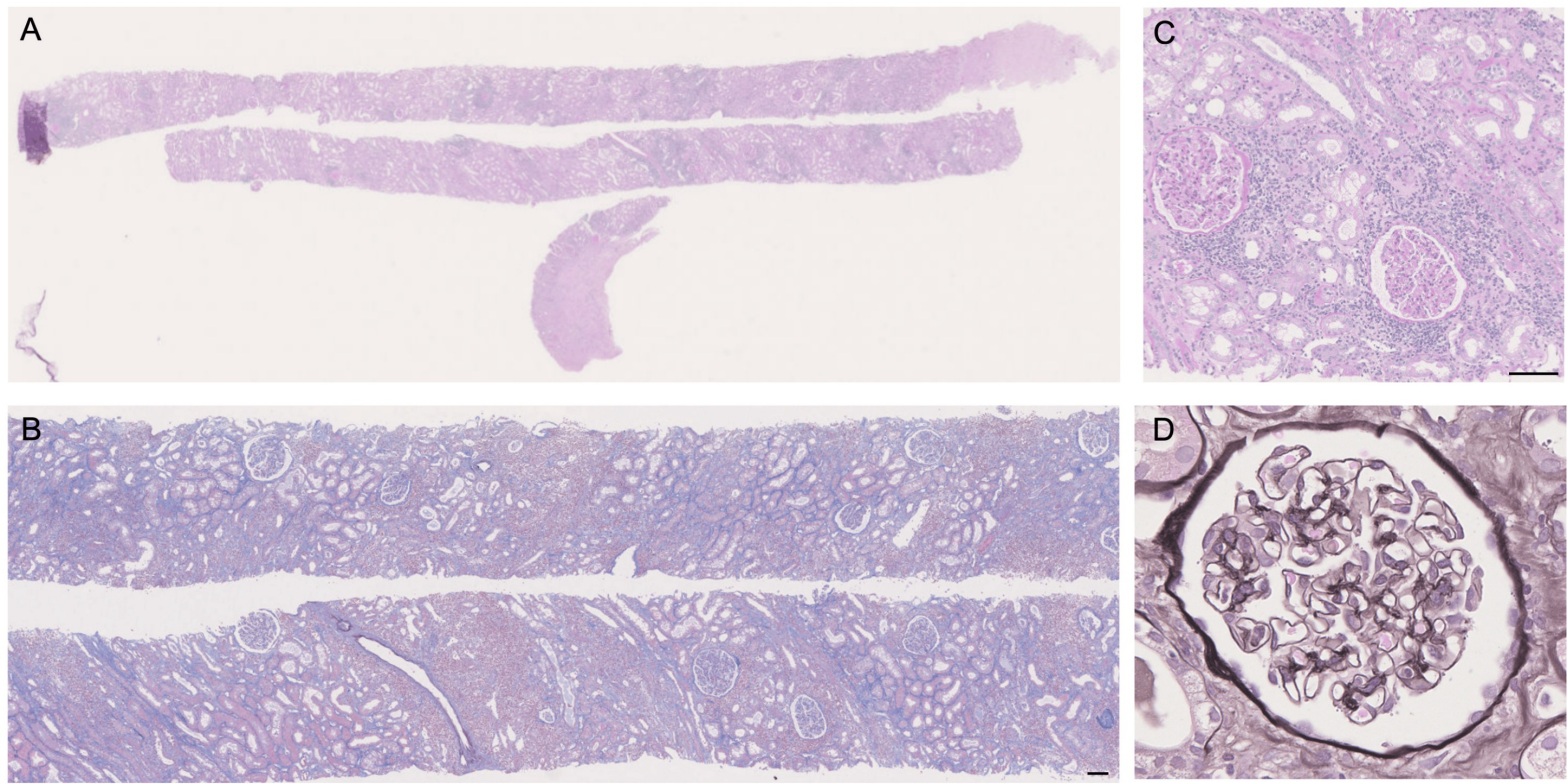
Histological findings of the second episode biopsy (A) Low-power view of the entire section (periodic acid-Schiff (PAS) staining); (B) Inflammatory cell infiltration observed in the interstitium (Masson’s trichrome staining), Scale bar =100 μm; (C) Tubulitis and interstitial inflammatory cell infiltration (PAS staining), Scale bar =100 μm; (D) Intact glomerulus (periodic acid–methenamine (PAM) staining).

In November 2023, an ABO-compatible living-donor kidney transplantation was performed using his mother as the donor. A protocol biopsy performed one hour post transplantation revealed no evidence of rejection. Immunosuppressive therapy was administered based on a standard protocol with steroids, basiliximab, mycophenolate mofetil (MMF), and tacrolimus. The clinical course during hospitalization is shown in Figure 1. On postoperative day 5, a roommate tested positive for COVID-19. Although asymptomatic, the antigen test result of the patient was positive, and he was treated with molnupiravir. Ten days later, the COVID-19 antigen remained detectable, and on postoperative day 15, the MMF dose was reduced from 1,500 mg/day to 1,000 mg/day. Serum creatinine initially improved to 2.41 mg/dL by postoperative day 7 but subsequently increased gradually. An episode biopsy was performed on postoperative day 19 (Figure 2). Thirteen glomeruli were observed, one of which was globally sclerotic. No crescents or adhesions, mesangial matrix expansion, intratubular proliferation, or basement membrane duplication were observed. Interstitial fibrosis and tubular atrophy (IFTA) involved approximately 10% of the cortex, with inflammatory cell infiltration affecting 20% of the cortical area. Inflammatory infiltration was confined to the non-fibrotic cortical interstitium, whereas IFTA areas were spared. Focal tubulitis was present in non-IFTA tubules but absent in IFTA regions. No endarteritis or peritubular capillaritis was observed. No clear FSGS lesions were identified. The pathological diagnosis based on the Banff classification was category 3 (borderline), suggesting acute TCMR. Following the biopsy, steroid pulse therapy was administered for three days starting on postoperative day 19. Serum creatinine initially improved to 2.6 mg/dL on day 26 but subsequently worsened. Everolimus (1.5 mg/day) was initiated on postoperative day 38; however, renal function continued to decline, reaching 5.86 mg/dL by postoperative day 41. A second episode biopsy was performed on day 41 (Figure 3). Thirty-six glomeruli were observed, including six globally sclerotic glomeruli and one fibrocellular crescent. No glomerulitis, mesangial matrix expansion, intratubular proliferation, or basement membrane duplication was observed. In the interstitium, mononuclear cell-predominant inflammatory infiltration involved approximately 50% of the cortical area, accompanied by severe tubulitis. Fibrosis and tubular atrophy were observed in approximately 30% of the cortical region. In the non-fibrotic cortical interstitium, inflammatory cell infiltration occurred in approximately 40% of the area, whereas IFTA regions showed inflammatory cell infiltration in approximately 20% of the interstitial space. No endarteritis was observed. No features suggestive of calcineurin inhibitor (CNI) toxicity were observed. Histopathological examination confirmed acute TCMR. The extent of TCMR was greater than that observed in the prior biopsy.

On postoperative day 41, the patient experienced a rapid decline in urine output and underwent two sessions of hemodialysis. Anti-human thymocyte immunoglobulin (ATG) at 100 mg/day was administered for nine days starting on postoperative day 40. Plasma exchange was not performed owing to ABO compatibility, the absence of donor-specific antibodies, and the previous biopsy findings consistent with TCMR. Renal function gradually improved, and the patient was discharged on postoperative day 57. 

**Figure 4:**
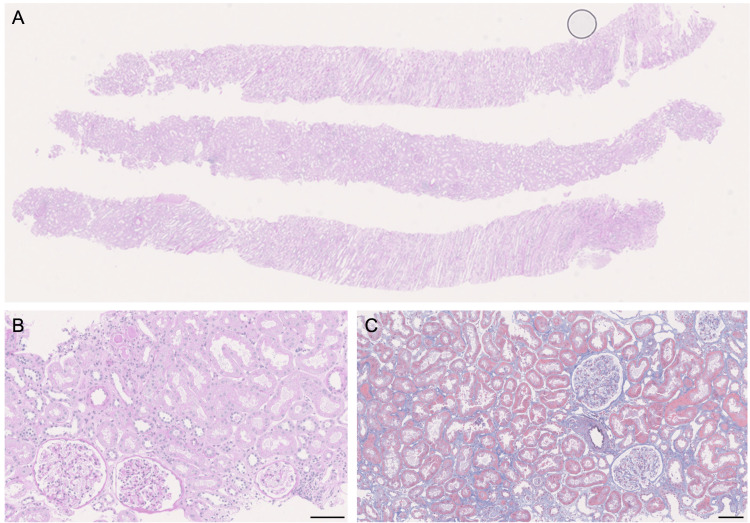
Histological findings of the three-month protocol biopsy (A) Low-power view of the entire section (periodic acid-Schiff (PAS) staining); (B, C) Interstitium with minimal inflammatory cell infiltration (B: PAS staining; C: Masson’s trichrome staining), Scale bar =100 μm.

A three-month protocol biopsy was performed (Figure 4), which revealed 12 glomeruli with no global sclerosis, crescents, or adhesions. No intratubular proliferation, mesangial expansion, or basement membrane duplication was observed. Interstitial inflammatory infiltration involved approximately 10% of the cortical area, with IFTA present in a similar proportion of the cortex. The non-fibrotic cortical interstitium showed minimal inflammatory infiltration, whereas IFTA regions exhibited approximately 50% inflammatory infiltration. Tubulitis was limited to atrophic tubules. No evidence of arteritis was noted. No findings indicated acute rejection or CNI toxicity.

**Figure 5:**
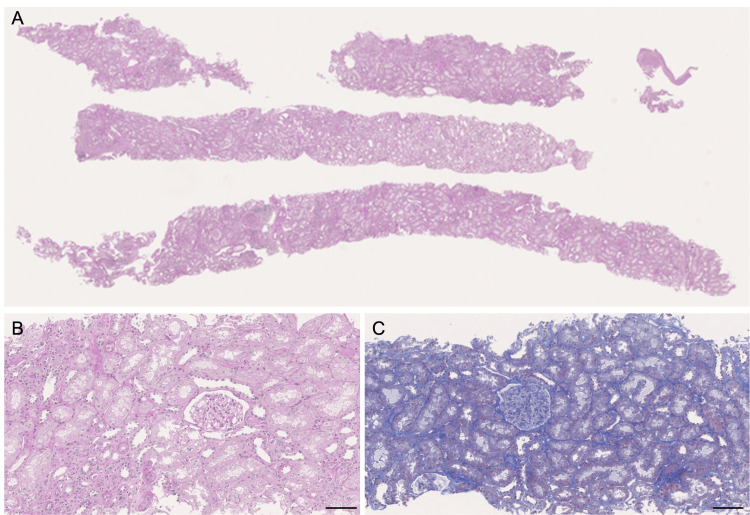
Histological findings of the one-year protocol biopsy (A) Low-power view of the entire section (periodic acid-Schiff (PAS) staining); (B, C) Interstitium with minimal inflammatory cell infiltration (B: PAS staining; C: Masson’s trichrome staining), Scale bar =100 μm.

A one-year protocol biopsy was performed (Figure 5), which revealed 11 glomeruli without global sclerosis, crescents, or adhesions. No intratubular proliferation, mesangial matrix expansion, or basement membrane duplication was observed. Inflammatory cell infiltration involved approximately 5% of the cortical interstitium, and tubular atrophy affected approximately 10% of the cortex. Minimal inflammatory infiltration was noted in the non-fibrotic cortical interstitium, while IFTA regions exhibited approximately 50% inflammatory cell infiltration. Tubulitis was limited to atrophic tubules. No evidence of arteritis was noted. No findings indicated acute rejection or CNI toxicity.

**Figure 6:**
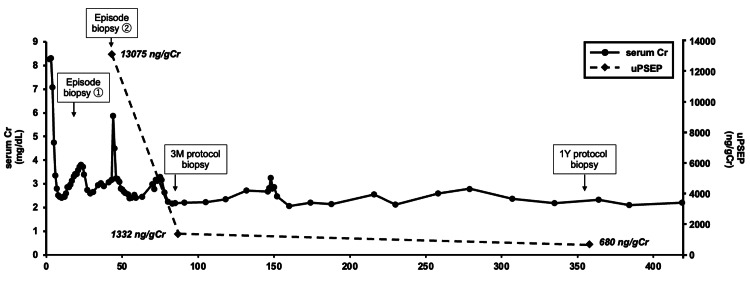
Changes in serum creatinine and urine preseprin Cr: creatinine; uPSEP: urinary presepsin; 3M: three months; 1Y: one year

Urinary presepsin levels are shown in Figure 6. At the time of the second episode biopsy with TCMR, the urinary presepsin level was 13,075 ng/gCr. At the three-month and one-year protocol biopsies, which showed no evidence of rejection, urinary presepsin levels decreased to 1,332 ng/gCr and 680 ng/gCr, respectively.

## Discussion

This report describes a kidney transplant recipient who contracted COVID-19 immediately after transplantation and subsequently developed TCMR following a reduction in immunosuppressive therapy. At the time of the second episode biopsy, which demonstrated severe TCMR, urinary presepsin levels were markedly elevated. In contrast, during the three-month and one-year protocol biopsies, when no histological evidence of rejection was observed, urinary presepsin levels remained low. These findings indicate that urinary presepsin, a non-invasive and rapid test, closely correlates with renal function and histological findings, suggesting its potential utility as a reliable marker of TCMR activity.

We have previously reported the diagnostic utility of urinary presepsin for detecting TCMR during kidney transplantation [10]. In our earlier analysis of 39 patients (three with TCMR and 36 without TCMR), the urinary presepsin values were 6788.63 ng/gCr in the TCMR group and 777.61 ng/gCr in the non-TCMR group. The receiver-operating characteristic curve for predicting TCMR showed a cutoff value of 3961 ng/gCr, with an area under the curve of 0.982. In the present case, urinary presepsin measured at the second episode biopsy, when TCMR occurred, was markedly above the cutoff, whereas values at protocol biopsies performed three months and one year later, when TCMR was absent, were below the cutoff. To date, no study has evaluated its usefulness for longitudinal assessment of disease activity. To our knowledge, this is the first report to demonstrate the potential of urinary presepsin for the longitudinal monitoring of TCMR, suggesting that it may serve as a reliable biomarker reflecting rejection activity.

In this case, both urinary presepsin and serum creatinine increased during rejection episodes. However, creatinine primarily reflects renal function and is not specific to rejection activity, whereas urinary presepsin appears to more directly reflect the histological activity of rejection. Among non-invasive urinary biomarkers for post-transplant rejection, CXCL9 and CXCL10 are the most extensively studied [5,6]. These chemokines act via CXCR3, recruiting NK cells, mononuclear cells, activated T cells, and B cells, and their urinary levels are elevated in both TCMR and antibody-mediated rejection (ABMR) compared with patients without rejection [5]. As treatment strategies differ between TCMR and ABMR, distinguishing rejection type is clinically important; however, CXCL9 and CXCL10 alone cannot reliably discriminate between subtypes. In this report and our previous study, we showed that urinary presepsin increases during TCMR [10]; however, its behavior in ABMR remains to be explored and warrants further investigation. Additionally, several confounding factors are known for CXCL9 and CXCL10, including bacterial urinary tract infections, BK polyomavirus infection, recurrent AA amyloidosis, and thrombotic microangiopathy [5]. Urinary presepsin has also been reported to increase in urinary tract infections [11]. Given that transplant recipients are at higher risk of urinary tract infections due to immunosuppressive therapy, careful clinical evaluation is required. We have previously shown that urinary presepsin increases in interstitial nephritis [9]; however, whether it is elevated in the conditions mentioned as confounding factors for CXCL9 and CXCL10 remains unclear and warrants further investigation. Regarding assay performance, CXCL9 and CXCL10 measurement using the Ella immunoassay requires approximately 1.5 hours [4], whereas presepsin can be quantified in just 17 minutes using a high-sensitivity chemiluminescent enzyme immunoassay. In Japan, presepsin is already established as a blood-based biomarker for sepsis, and this system can be readily adapted for urinary measurement in clinical practice. Although multiple retrospective studies have shown a correlation between urinary CXCL10 and kidney allograft rejection, the randomized controlled trial failed to demonstrate a benefit of urinary CXCL10 monitoring on one-year outcomes [12]. To improve predictive accuracy, multivariable models combining multiple biomarkers are being investigated [3]. Urinary presepsin may serve as a complementary marker, potentially enhancing the accuracy of rejection risk assessment and post-transplant monitoring.

This study had some limitations. First, as presepsin is an infection-related marker, careful distinction between rejection and infectious inflammation, such as urinary tract infection, is essential. Second, given that presepsin likely reflects tubulointerstitial inflammation [9], elevated presepsin levels may occur in cases of tubulointerstitial nephritis independent of rejection. Finally, as this report describes a single case, larger multicenter studies are required to establish robust cut-off values and validate their clinical utility. In the future, prospective validation studies or large cohort evaluations, including comparisons with CXCL9 and CXCL10, should be considered.

## Conclusions

We report a case of a kidney transplant recipient who developed TCMR following a reduction in immunosuppressive therapy due to COVID-19 infection immediately after transplantation. Urinary presepsin is a noninvasive and rapidly measurable biomarker that allows repeated assessments. It closely correlates with renal function and the histological findings of TCMR, suggesting its potential as a reliable indicator of rejection activity and its usefulness for longitudinal monitoring of TCMR. Larger multicenter studies are needed to establish its diagnostic thresholds and clinical role.
